# Characterization and Typology of Hunting Dog Packs (Rehalas) and Breeder Management Practices in a Mediterranean Mountain System

**DOI:** 10.3390/ani16040572

**Published:** 2026-02-12

**Authors:** Carlos Poderoso Martínez, Ana González-Martínez, Manuel Luque Cuesta, Evangelina Rodero Serrano

**Affiliations:** 1 Doctoral Program in Natural Resources and Sustainable Management, University of Córdoba, 14071 Córdoba, Spain; poderoso967@gmail.com; 2 Animal Production Department, University of Córdoba, 14071 Córdoba, Spain; pa1rosee@uco.es; 3 Royal Spanish Federation of Livestock Purebred Associations (RFEAGAS), 28001 Madrid, Spain; manuel.luque@rfeagas.es

**Keywords:** traditional hunting practices, socio-demographic profile, Large-size Podenco Andaluz breed, hunting management, hierarchical clustering

## Abstract

This study aimed to characterize hunting dog packs (rehalas) and identify management typologies within a Mediterranean mountain system (Sierra Morena region of Córdoba). An ethno-demographic survey was designed and completed by 30 breeders. Descriptive statistics were used for general characterization, while variability assessment and typology identification were performed using multiple correspondence analysis and hierarchical clustering. The typical dog pack breeder was a 48-year-old man with extensive experience (28.5 years) and basic formal education. Dog packs comprised an average of 51.9 dogs, predominantly of the Large-sized Podenco Andaluz breed, participating in approximately 40 hunting events per year. Feeding practices commonly combine commercial feed with supplementary food items. Health management included routine deworming every six months, and 43% of breeders reported concern about leishmaniasis. Training generally began at around 14.5 months of age and followed regular weekly routines. Ten factors explained 82.4% of the observed variability, allowing the identification of three typologies: traditional, pragmatic, and non-organized. These findings underline the cultural, genetic, and socio-ecological relevance of dog packs as working groups in Mediterranean rural systems. The long-term sustainability of these systems depends on reinforcing selective breeding, improving health management, and safeguarding traditional practices adapted to each identified typology.

## Simple Summary

This study describes how hunting dog packs in the Sierra Morena region (Andalusian, Spain) are organized and managed by their owners. Information was collected through surveys with breeders, allowing the identification of different ways of managing dog packs based on aspects such as pack size, breeding, health care, feeding, and training practices. The findings highlight the cultural, genetic, and social importance of dog packs in Mediterranean rural areas. Preserving their long-term viability will require supporting responsible breeding, improving health management, and protecting traditional practices adapted to the specific characteristics of each management group. Policies that recognize this diversity are essential to conserve this unique heritage and sustain its contribution to local socio-economic development. The findings of this study should be interpreted within the specific context of the Sierra Morena region. This regional focus allows for an in-depth characterization of a particular hunting system while highlighting the need for caution when extrapolating the results to other regions or hunting contexts with different environmental and cultural characteristics.

## 1. Introduction

Hunting is a multifunctional activity [1] and an important source of income in many regions of the world, particularly where agriculture and forestry are less profitable [2]. In Spain, it is a popular outdoor practice that generates benefits, which may help counteract rural depopulation and contribute to wildlife conservation through the management and rational use of natural resources [2].

Since their domestication, dogs have accompanied humans in their daily activities, hunting being one of them. The types of dogs used in this game-related activity are highly diverse and directly dependent on the hunting modality as well as the target species. The multitude of dog breeds that exist today is the result of different domestication events, together with the selective pressure applied and the accumulation of deleterious mutations associated with artificial selection for various functions [3]: locating prey (trackers), retrieving downed game (retrievers), restraining wounded but still-living prey (grip or holding dogs), etc.

In Europe and the Americas, hunting driven by dogs remains a prominent form of hunting, targeting mainly ungulates but also medium and large-sized carnivores [4,5,6]. In the Iberian Peninsula and France, wild boar (*Sus scrofa*) and red deer (*Cervus elaphus*) hunting has a long and deeply rooted tradition [7]. In Spain, one of the most traditional modalities for hunting these species is the “Montería” which was first regulated in 1180 CE [8], and later became, from the Middle Ages (13th and 14th centuries) through the 19th century, an exclusive privilege of monarchs and the nobility [9].

Currently, 85% of Spain’s territory is designated as hunting land. However, commercial hunting is more common in the southern region of the country, particularly in Andalusia, where a large proportion of hunting licenses are issued [8] and substantial numbers of wild boar and red deer are harvested [10].

The Hunting Law enacted in 1970 [11] defined the Private Hunting Estate as a continuous land area suitable for hunting use, which must be declared and recognized by the competent authority. This model has become predominant throughout Spain, particularly in the Sierra Morena region [12]. The hunting area of this mountain system covers 437,852 hectares in the province of Córdoba (Andalusia). It includes two protected areas [13]: the Hornachuelos and Cardeña-Montoro Natural Parks. These districts account for 72% of the total value of hunted game in the province, and the sale of hunting posts contributes 11% of the total agricultural production value of the region; additionally, the commercialization of game meat has significant economic importance [14].

The substantial annual revenues generated by this activity (90 million euros) result from both the sale of hunting posts and the payments made to the dog pack owners or breeders (rehaleros), who are responsible for systematically searching the hunting area with their dogs and driving the game toward the hunters [15]. Dogs are the central actors in this practice, and the breeders are tasked with ensuring their welfare, with the animals being treated with respect and appreciation by the hunters.

This hunting modality involving dogs generates both direct and indirect benefits. In each driven hunt, a variable number of hunters (20–75) participate, positioning themselves at fixed locations within the hunting estate, known as shooting posts. Dog packs also play a key role; they often remain concealed among the forest and shrubs until they intervene in detecting the prey, driving it toward the hunters, and holding wounded game for its final dispatch if necessary [16]. In some hunting contexts, breeders may manage groups of approximately 15–30 dogs [8], although the size of hunting dog packs can vary widely depending on local traditions, available facilities, and the characteristics of the hunting area.

The status of dog pack owners is formally recognized in hunting regulations, which allows them to participate in hunts and carry the authorized bladed weapons used to dispatch wounded or restrained animals, as well as a blunderbuss loaded with blank ammunition [17]. They are responsible for directing the dogs toward the prey and for killing or dispatching with a knife the animals wounded or downed by the hunters, but they are strictly prohibited from using firearms during the hunts. Individuals engage in this activity either as a primary profession, a secondary occupation, or for leisure. They are not only responsible for handling the dogs during hunting days, but also for breeding, selecting, and training the dogs used in hunting [8].

Despite the importance of big-game hunting in Sierra Morena, the activity of breeders and their dog packs remains largely unknown, with very few studies addressing this topic. Recently, Sánchez-García and Villanueva [8] published the first analysis of the sociodemographic characteristics of dog pack breeders (rehaleros) in Spain. However, significant knowledge gaps persist regarding the types of dogs used in hunting and the factors that shape the differentiation of dog packs. Therefore, the present study aimed: (i) to analyze the socioeconomic, ethological, and functional characteristics of dog packs in Sierra Morena of Cordoba (Spain), in relation to their management and environmental context; (ii) to identify potential differentiated typologies; and (iii) to determine the main systems and factors that condition and constrain this productive sector.

## 2. Materials and Methods

### 2.1. Study Design

A survey was designed and conducted in the form of interviews. The dog pack breeders were located and contacted through the mediation of hunting federations and dog pack associations. The sample was later expanded using a snowball sampling approach [18]. Attendance at the annual “Intercaza” fair in Córdoba also facilitated participant recruitment. Due to the considerable amount of time required to complete the full questionnaire, part of each interview was conducted in person, through direct conversations with the breeder at the location of the dog pack, and later completed by telephone. The survey included 50 questions (70% open-ended) addressing the following topics: (i) information on the location of the dog pack, its environment, and hunting grounds; (ii) the number and types of animals composing the dog pack (age, breed, and sex), including their regular activity (target prey species and number of hunts per season) and function (tracking, running, or holding); (iii) selection and preparation of the animals (selection of breeding stock, criteria for choosing dogs for each hunting event, training practices, and whether dogs are sterilized); (iv) economic aspects and types of services provided by the dog pack, including whether animals are sold to other dog packs; (v) social characteristics of the dog pack owner (age, sex, educational background, occupation, years of experience, etc.) as well as whether they receive assistance in caring for the dog pack; and (vi) animal care practices (health management and feeding regime).

The time devoted to each interview was not predetermined to encourage a fluid and friendly conversation [19]. The interviews were conducted during the spring and summer of 2022, 2023, and 2024, coinciding with the closed hunting season, when breeders and their dog packs are resting. Participation in the study was voluntary, and anonymity was guaranteed to all interviewed in the publication of the results. The survey was answered by 30 dog pack breeders, a sample size considered appropriate given the restricted accessibility of this social group and the exploratory nature of the study.

### 2.2. Characteristics of the Study Area and Location of the Dog Packs

Sierra Morena covers an area of just over one million hectares, of which approximately 339,000 ha belong to the province of Córdoba. The region is characterized by a subtropical Mediterranean climate, with average temperatures ranging between 15 and 20 °C and annual precipitation of 600–890 mm. The vegetation consists of Mediterranean scrubland, including several Quercus species (holm oak, cork oak, and Portuguese oak), along with low shrub formations [20].

**Figure 1:**
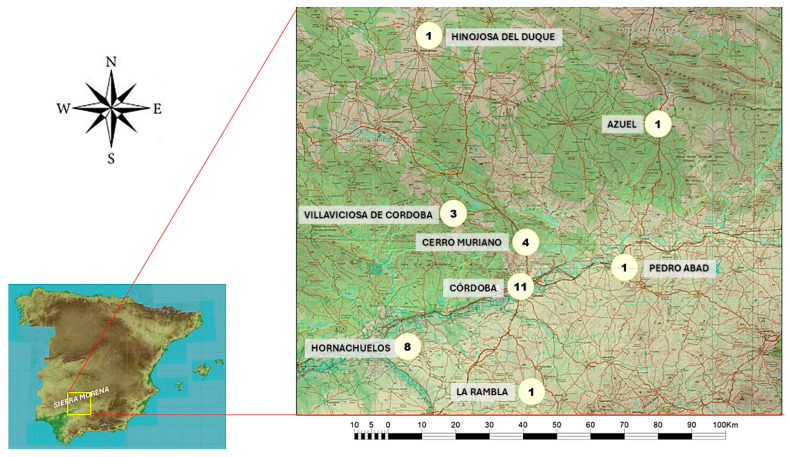
Geographic location and number sampling dog packs in Sierra Morena, Córdoba (Spain). Source: Author’s own.

The thirty interviewed breeders kept their dog packs in the municipalities of Córdoba (11), Hornachuelos (8), Villaviciosa de Córdoba (3), Cerro Muriano (4), Azuel (1), Hinojosa del Duque (1), La Rambla (1), and Pedro Abad (1), and carried out their hunting activities in Sierra Morena area (Figure 1).

### 2.3. Statistical Analysis

In an initial stage aimed at characterizing the dog packs, 48 variables were analyzed. For quantitative variables, mean values and measures of variation were obtained, while absolute and relative (%) frequencies were calculated for the remaining variables.

To construct the typology and characterization of the dog packs, variables were selected based on criteria such as a coefficient of variation greater than 60%, lack of correlation among variables, and non-linear dependence [21]. Multiple correspondence analysis (MCA) was applied to reduce the number of variables and synthesize most of the data variability [22]. Multiple correspondence analysis (MCA) was performed using Statgraphics Centurion version XVI.1, which allows the inclusion of both categorical and quantitative variables. Quantitative variables were internally standardized and treated by the software to contribute to the overall inertia of the data structure without requiring prior manual categorization. The analysis was exploratory and aimed at summarizing the joint variability of all variables rather than modeling a response variable. Based on the partial correlation matrix and initial MCA models, the number of variables was reduced to 28. Ten principal factors were selected, and an orthogonal varimax rotation was applied to better associate the selected variables with the extracted factors. Model adequacy was assessed using Bartlett’s test of sphericity and the Kaiser–Meyer–Olkin index (>0.7) [23].

In the second stage, the dog packs were classified using hierarchical cluster analysis based on Ward’s method, employing squared Euclidean and Manhattan distances. The optimal number of clusters was determined using the Elbow method [24], selecting the solution that yielded the highest percentage of correctly classified cases and the greatest differences among the original variables. Comparisons between clusters were performed using analysis of variance for quantitative variables and the χ^2^ test for qualitative variables (*p* < 0.05).

Statistical analyses were performed using Statistica 12.0 for Windows and Statgraphics Centurion versión XVI.1. software.

## 3. Results

### 3.1. Characteristics of the Dog Pack in Sierra Morena

Table 1, Table 2, Table 3 and Table 4 present the characteristics of dog packs, their owners, and management practices related to dog care and hunting activities.

#### 3.1.1. Socioeconomic Characteristics of the Dog Pack Owner

The dog pack breeders were men with an average age of 48 years (±2.21 SE) and 28.50 (±2.32 SE) years of experience. Most had basic education (79.9%), while only a small proportion had vocational training (10.0%) or university education (6.7%).

Each dog pack employed an average of 1.4 caretakers (±0.11), who were responsible for daily tasks. These duties were carried out mainly by the breeder, who, in most cases (73.33%), received assistance. In 36.67% of cases, the assistance came from family members (such as a spouse), particularly among younger breeders. In other cases, support was provided by colleagues from their other jobs (26.67%) or by an assistant (6.67%). Only 3.33% of respondents reported hiring paid employees to perform these tasks.

Regarding the territories in which the dog packs operated, we found that their main activity occurred in hunting estates located near their facilities within the province of Córdoba. However, in four cases, the dogs traveled considerably longer distances to participate in hunts conducted either in the Sierra Morena region, originating from the province of Ciudad Real, or in Los Alcornocales Natural Park (Cádiz). For this reason, although dog packs attended an average of 39.67 (±4.25) hunts per hunting season, variability in this figure was very high (CV = 58.73%). The number ranged from dog packs who participated in only five or ten hunts per year to others who carried out up to 130.

#### 3.1.2. Care and Management of the Dog Pack

The dogs were trained for approximately three hours per day, three times per week, to be prepared for the hunting season. For training purposes, the dog pack breeder grouped the dogs either by age or hunting group. The composition of each hunting group was determined by the type of hunt, the characteristics of the terrain and target species, and the specific dog packs scheduled to participate on each hunting day.

The animals were fed daily, either exclusively with commercial feed (26.67%) or with a combination of commercial feed and other food items such as meat, game remains, table scraps, and bread (70%). It was uncommon for a dog pack to be fed solely on meat and bread (3.33%). Another task carried out by the breeder or assistant was the daily cleaning and disinfection of the facilities to maintain them in good condition and prevent leishmaniasis. Due to the high incidence of leishmaniasis that the respondents’ dogs had experienced at some point (43.33%), 50% of them implemented measures to control it. These included the use of protective collars (10%), thorough cleaning and disinfection of the facilities (33.33%), and, in some cases, the use of insect traps (6.66%).

As prophylactic measures against other pathologies, the dogs were dewormed every 6.07 months (±0.47) and vaccinated annually against rabies. In addition, when they were puppies, they received the appropriate vaccinations, including those against distemper and parvovirus (90%).

The dog packs specialized in hunting ungulates (86.67%), rabbits (6.67%), and both rabbits and wild boars (6.67%). Each breeder kept an average of 51.87 (±3.49) dogs, of which 39.57 (±3.02) were males and 12.23 (±0.81) were females.

Each dog pack consisted of just over fifty dogs, 48.63 (±3.30) of which were capable of tracking and pursuing game, while an average of 16.70 (±4.14) animals were responsible for holding wounded prey. The dog packs were largely self-sustaining in terms of dog production, although 73.33% of respondents reported having introduced external genetics at some point, either within the past five years (45.45%) or more than five years ago (31.82%).

Dogs began training once they were slightly over 10 months of age (10.45 ± 0.67). A total of 96.67% underwent training nearly three days per week, conducted by the dog pack owner (100%) to prepare them for the hunting season. Breeders indicated that training does not involve the deliberate use of external rewards or punishment. Training groups were formed according to dog age (40.00%), hunting group assignment (46.67%), or the specific pack configuration in which the dog was expected to perform.

At the beginning of the hunting season, the breeder established what were known as dog pack teams (86.67%), selecting dogs according to the type of hunt, terrain, or target species. Furthermore, the subset of dogs that would participate in each hunting day was chosen using the same criteria as for team formation (16.67%), based on personal criteria (56.67%), or considering the dog’s fatigue (16.67%) after recent participation in another hunt. Sixty percent of respondents stated that they collaborated with other dog packs.

#### 3.1.3. Types and Selection of the Animals

The predominant dog type was the large-sized Podenco Andaluz, commonly known as Podenco Campanero (83.34%), although most dog packs also included a few tracking or holding animals (13.33%). In some cases, breeders crossed the Podenco Campanero with the Spanish Mastiff (6.66%) to obtain a more multifunctional animal. However, a considerable proportion (23.33%) preferred keeping dogs capable of tracking, pursuing, and holding prey.

During hunting days, dog pack owners showed a preference for male dogs (50%) or females (16.67%), while the remaining 33.33% were indifferent to the sex of the animals. However, non-breeding males (86.67%) were generally not sterilized, and under no circumstances were females in estrus taken to the hunt.

There was considerable diversity in the criteria used to select breeding animals. Morphological traits were generally considered globally across body parts (i.e., overall type = 40%), either as isolated criteria or in combination with coat color or other traits (e.g., overall type, coat, and size = 3.33%). Breeders also considered functional criteria when choosing breeding stock, assigning primary importance to traits such as endurance, intelligence, and holding ability (16.67%); endurance and latency, or sustained performance during work (16.67%); strength and obedience (3.33%); or excellent olfactory capacity (16.67%), among others.

In addition to external and functional traits, breeders also considered the dog’s temperament (93.33%), with nobility (understood by breeders as a calm, balanced, and reliable behavior during hunting activities and in interactions with humans and other dogs) being the most valued trait (53.33%), whereas distrust (3.33%) or maternal aptitude (3.33%) carried far less weight in breeding decisions. However, the final decision was typically made after observing the dog’s performance during hunting days, as the animal was not definitively used as a breeder until nearly two hunting seasons had passed (1.83 ± 0.20).

#### 3.1.4. Location and Characteristics of the Facilities

The dog packs were located in secluded areas (at the end of tracks, within private estates, etc.) but not far from urban centers, due to the daily care that the animals required. The dogs were housed in rectangular or square buildings that provided a sheltered resting area protected from adverse weather conditions. Inside these facilities, the animals were either individually tied or accommodated in kennels, where they were housed in small groups to prevent potential aggression.

Long troughs were used to supply water ad libitum to all animals, allowing easy water replacement and cleaning. In addition, the facilities typically included an outdoor area used as a recreational yard for the dogs, whose size varied depending on the characteristics and scale of each dog pack.

### 3.2. Analysis of Dimensions Determining Variance Among Dog Packs

The total variability of the dataset, considering all 28 management, demographic, and functional variables included in the multiple correspondence analysis, was explained by 27 factors or dimensions, with the first 10 factors accounting for 82.37% of the total variance (Table 5). The first factor explained 16.40% of the variance and was associated with the number of hunts, the number of animals (size of dog pack), and the type of dog.

The second factor explained 13.61% of the variance and was primarily influenced by the hunting area and the age of the dog pack owner. The third factor accounted for 9.87% of the variance and was represented by training duration. The fourth factor (8.58%) was associated with the animals’ diet and the predominant prey species in the hunting estates where the hunts occurred. The fifth factor (7.96%) reflected the geographical location of the dog pack. Factors 6 and 7 were related to the dogs’ deworming schedule and collaboration with other dog packs, explaining 6.55% and 6.23% of the total variance, respectively.

The eighth factor accounted for 5.31% of the variance and was mainly driven by the temperament-based criteria used by breeders when selecting future breeding animals; it was also influenced by whether dogs trained in groups formed according to the hunting teams used during the hunts. Finally, factors 9 and 10 (4.63% and 3.22%) were associated with the incidence of leishmaniasis and the number of training days per week, respectively.

### 3.3. Cluster Analysis for the Identification of Typologies Among Dog Packs

**Figure 2:**
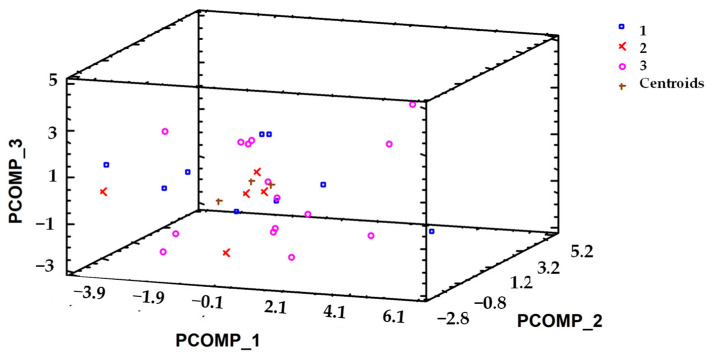
Cluster interaction and MCA dimensions of dog packs in Sierra Morena, Córdoba.

**Figure 3:**
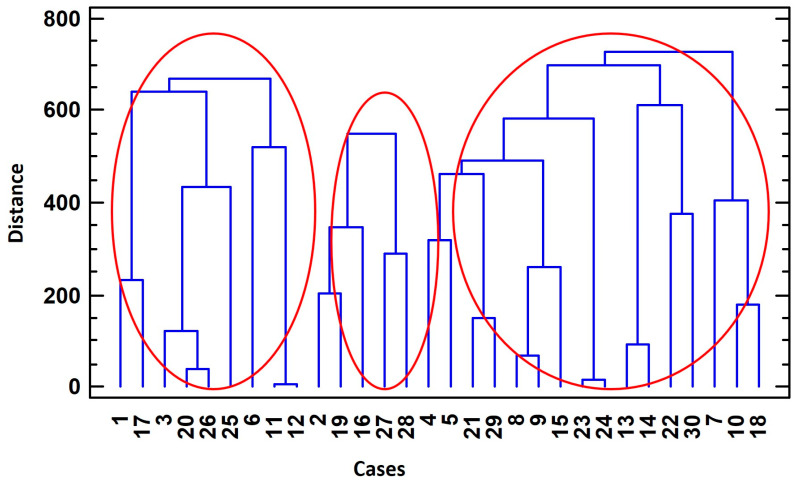
Clusters of dog packs in Sierra Morena, Córdoba. Red circles indicate the different typologies identified in the cluster analysis.

Hierarchical cluster analysis performed on the MCA scores identified three breeder profiles based on overall patterns of management, experience, and organization. These profiles should be interpreted as exploratory typologies reflecting dominant combinations of practices rather than as statistically distinct groups. While most individual variables showed overlapping distributions among clusters, differences in deworming practices were more consistently observed (Table 6). The remaining variables contributed to the multivariate structure of the clusters through their combined effects rather than isolated statistically significant differences. The distribution of dog packs among these three types was determined by six variables from those considered, which are integrated into three of the principal components or factors (Figure 2). The first cluster (Cluster 1) comprised 30% of the dog packs (9), Cluster 2 included 16.67% (5), and Cluster 3 contained 53.33% (16) (Figure 3). CP_1 is the factor with the greatest weight in the grouping of the dog packs included in Cluster 2. CP_2 characterizes the dog packs that form Cluster 3. CP_3 was the factor with the highest contribution to the formation of Cluster 1.

Cluster 1 (traditional dog pack) comprises nine dog packs located mainly in two areas: Hornachuelos and the surroundings of the city of Córdoba. These breeders had an average age of 50.67 ± 4.63 years and tended to approach their activity from a more traditional perspective, characterized by long-term experience, reliance on family-based knowledge transmission, and the maintenance of customary management and training practices. They placed high value on the esthetic quality and homogeneity of their packs, basing breeding selection on overall type and, in some cases, coat color.

The dog packs consisted of approximately 45 dogs, of which 27% were females. Dogs trained for three hours a day, slightly more than two days per week, beginning at nearly one year of age. Each dog pack attended around 33 hunts per year and was managed by the owner, who, in half of the cases, received assistance from a helper. In terms of health management, dogs were dewormed every six months, vaccinated as puppies, and annually against rabies. Despite the incidence of leishmaniasis, only half of the dog packs implemented minimal preventative measures against this disease.

Cluster 2 (pragmatic dog pack) was the smallest of the three groups, comprising only five dog packs, and represented a specific configuration of management practices identified through the multivariate analysis rather than a statistically distinct group. They reported collaborating with other dog packs, reflecting a cooperative approach to hunting activities. They were less concerned with traditional aspects: 60% formed their hunting group on purely practical grounds, and 60% had no preference for the sex of the dogs, selecting instead those that performed best during the hunt. They even included tracking dogs in the hunting team to improve the overall performance of the group.

Their selection criteria were based more on functionality than on esthetics (such as type or coat color), and they were primarily interested in the results obtained from their hunting activity. Most dog packs were economically self-sustaining and exchanged animals with other breeders. The dog pack size was like that in Cluster 1, but daily maintenance tasks were managed by a single person.

They participated in 36 hunting sessions per year, for which the dogs were prepared with training sessions starting at around eleven months of age, lasting three hours and carried out nearly four days per week. Training was sometimes performed individually rather than with the entire pack. Dogs received antiparasitic treatment every nine months and were vaccinated as puppies, with annual rabies vaccination as well. Regarding leishmaniasis, the incidence was low, although preventive measures such as cleaning and disinfection are routinely applied.

Finally, Cluster 3 (non-organized dog packs), composed of 16 breeders, represented a broad and heterogeneous group that does not appear to be governed by the rules of any specific organization or identifiable collective. One reason for this was the geographical diversity within the group: Córdoba and its surroundings (56.25%), Hornachuelos and Villaviciosa (18.75%), and Azuel, Hinojosa del Duque, La Rambla, and Pedro Abad (25%). They also varied widely in age, ranging from very young individuals to others with long-standing experience; in many cases, they inherited the dog pack from their parents and assisted them in daily tasks (43.75%). The average age was 46.06 years, but it included very young breeders (25 years old) as well as individuals close to retirement (64 years old).

The dog packs hunted in the province of Córdoba and in Ciudad Real and Cádiz, requiring longer travel distances for each hunting day. Nevertheless, only half of the breeders reported collaborating with other dog packs.

Regarding selection criteria, there was considerable diversity, as each breeder models their dog pack according to the modality, which they considered ideal. Consequently, breeding decisions were based on overall type, regional type, coat color, and size, as well as behavioral traits such as docility and bravery. These breeding practices were shared with other breeders, given that animals were exchanged and external genetics have recently been introduced (68.75%).

Cluster 3 also included a larger dog pack (57.81 dogs ± 5.39), and consequently, they participated in a greater number of hunts per year (44.81 ± 6.82). Daily care was carried out by the dog pack owner, although in half of the cases, they received assistance from another person. Dogs began training when they were slightly over nine months old, for less than 3 h per day, three days per week. Animals received antiparasitic treatment every 5 months; however, vaccination practices were inconsistent: some were vaccinated only against rabies, others vaccinated puppies and administered the mandatory rabies vaccine, and some vaccinated both puppies and adults, following the current legal requirements. Regarding leishmaniasis, nearly half of the total interviewees reported being significantly affected by the disease, implementing preventive measures such as collars, along with cleaning and disinfection of facilities and animals.

## 4. Discussion

In 2023, the first study examining the sociodemographic characteristics, habits, and perceptions of Spanish dog pack breeders was published [8]. In our study, we focused specifically on those from the province of Córdoba (Spain), whose dog packs are located in the Sierra Morena mountain system. Our findings provide detailed insights into the sociocultural, economic, and zootechnical realities of dog pack owners in this region.

Managing a dog pack is traditionally an activity passed down from parents to children. As a result, dog pack breeders typically gain experience from a very young age and progressively take on more responsibilities. In some cases, they co-manage the dog pack with their elders before eventually inheriting it or establishing their own [8]. The average age of breeders in Sierra Morena was 48 years. We also interviewed very young owners (<35 years). Sánchez & Villanueva [8] reported breeders as young as 18, although nearly half were older than 46, despite the physical demands of hunts, which require walking through dense forest and carrying harvested game. This is a lifelong activity, continued as long as physical condition allows.

Despite the lack of higher or specialized training, breeders from Cordoba perceive themselves as professionals, although most maintain their dog pack as a secondary occupation. Sánchez & Villanueva [8] highlighted that a portion of them were formally self-employed, and given the remuneration received in many hunts, the activity may be regarded as professional under fiscal regulations. Assistance in daily dog management is common and often provided by family members, particularly spouses, reflecting an ongoing modernization of the sector and the increasing presence of women in hunting activities [25].

The activity of dog pack breeders is shaped by strong seasonality, informality, and high maintenance costs [8]. Slightly fewer than half of them indicated that their dog pack is economically self-sustaining through income generated during the hunting season. The average annual expenditure is around €10,000, with approximately half corresponding to dog care and breeding and 27% to transportation to hunts [2].

According to testimonies from breeders collected during the survey, hunting with dog packs is perceived as a traditional activity strongly embedded in local culture, where participation is often motivated by enjoyment and continuity of customary practices rather than strictly economic considerations. Several breeders reported adjusting their service conditions to facilitate participation in hunting events, extending their hunting range beyond Córdoba to Ciudad Real and Cádiz. However, the number of hunts per year is mainly determined by the number of authorized hunting days (October–February), which ranges from 10 to 120 [8], and by dog pack size, which affects dog rotation and recovery.

There is currently a climate of secrecy surrounding dog packs, particularly in relation to their public visibility. Concerns expressed in public discourse regarding noise and smells associated with dog pack facilities have contributed to their location in remote areas, such as the end of secondary tracks or within private estates, away from public view. In this context, educational and outreach programs highlighting the ecological role of dog pack hunting, combined with clear animal welfare standards, could help improve social understanding of the activity and reduce the need for concealment.

In Andalusia, dog packs are regulated under legislation for zoological nuclei. Although a minimum indoor space per dog is not specified, an outdoor exercise area meeting the needs of all animals is required, and facilities must meet sanitation and hygiene regulations [26,27]. The dogs are generally housed in buildings providing shelter from adverse weather. Inside, animals may be kept individually tied or in kennels, in accordance with welfare regulations ensuring freedom of movement and easy access to resting and elimination areas [28,29].

The primary breed used in hunts in central and southern Spain is the Large-size Podenco Andaluz, known as Podenco Campanero, which has been associated with hunts for decades, leading to functional selection during the second half of the 20th century [30]. The Podenco Campanero is valued for its excellent scenting ability and endurance. Purebred individuals are preferred to facilitate the consolidation of desirable traits [31]. In border regions such as Jaén, Ciudad Real, and Extremadura, this Podenco is sometimes crossed with the Spanish Mastiff to obtain stronger and faster dogs [30]. Historically, holding dogs were common [32]; we found only one or two per dog pack, which were used to immobilize wild boar when required. Current holding breeds include the Spanish Alano, Dogo Argentino, Pitbull, American Staffordshire Terrier, and crosses thereof. Their limited number reflects the primary function of the dog pack, which is to drive game toward hunters, who are responsible for shooting [33]. Tracking dogs, such as small-sized hounds, are also included to guide and stimulate the Podenco Campanero group.

To ensure full functional efficiency during hunts, dog pack breeders train their dogs before each hunting season [34]. During the training, desired hunting behaviors are primarily shaped by innate predispositions linked to selective breeding and reinforced through experience during hunts, including social learning from more experienced dogs. The hunting activity itself is described by the breeders as intrinsically rewarding for the animals. Training intensifies as the season approaches and follows criteria passed down through generations or adopted from colleagues. Training groups are formed based on age and expected hunting-team configuration. Dogs that regularly participate in hunts are not used as breeders to avoid injury to reproductively valuable animals.

Animal selection is guided by oral tradition, long-term experience (trial and error), and continuous field observation. A matrilineal system prevails, with each dog pack maintaining some high-quality females. For each mating, the breeder selects the male deemed most suitable to optimize performance in the field while preserving preferred external traits. The most influential criteria include general and regional breed type, followed by size and coat. However, limited knowledge of genetic improvement and selection techniques may restrict functional progress and overall management efficiency. Breeders are often reluctant to share the genetics of their dogs, which are considered the product of many years of work, which partially explains the limited exchange of animals.

From a health standpoint, Andalusia, and particularly Córdoba, shows high seroprevalence of leishmaniasis [35]. Preventive strategies mainly rely on facility cleaning and disinfection, whereas repellent collars are used less frequently. In addition, breeders must comply with mandatory rabies vaccination in adult dogs and administer preventive vaccination to puppies.

The three identified typologies (traditional, pragmatic, and non-organized) do not represent rigid or mutually exclusive categories, but rather gradients of management strategies that coexist within the studied population. The absence of statistically significant differences for most individual variables reflects the shared cultural and functional framework of dog packs, while the typologies capture broader organizational tendencies. Group formation was influenced primarily by traditional breeding and selection practices, hunting areas, and economic factors. Traditional typology includes dog pack breeders strongly focused on maintaining longstanding organizational models and are reluctant to innovate or share genetic material. They possess extensive forest and wildlife knowledge, largely acquired through long-term experience and intergenerational transmission within family and local hunting networks [36]. Breeders from pragmatic typology have a more open attitude, collaborate with other dog packs, and integrate both traditional and contemporary practices. Non-organized typology is the largest and most heterogeneous, representing breeders who adapt more readily to current contexts, including younger participants. These dog packs tend to be larger, facilitating animal rotation when participating in hunts outside Córdoba.

Hunters in general, and dog pack breeders in particular, play an important role in managing hunting territories, contributing to the regulation of ungulate densities, reducing pressure on forest regeneration, mitigating crop damage, and influencing ecosystem structure [37]. Their activity also helps maintain agroforestry mosaics through the upkeep of firebreaks, forest paths, traditional routes, and pastoral areas [38]. Hunting additionally contributes to wildfire prevention [39], a significant concern given the severe impacts of fires in the Iberian Peninsula [40]. Combined with the broader conservation role of hunting [37], this underscores the continued relevance of dog pack breeders today.

Given the exploratory nature of the multivariate analyses and the restricted accessibility of the study population, the sample size was considered adequate to identify major patterns and management typologies, although the results should not be interpreted as statistically generalizable.

## 5. Conclusions

This study demonstrates that hunting dog packs in Sierra Morena (Córdoba) are located within protected natural areas, occupying secluded sites while remaining sufficiently close to urban centers to allow daily dog management. These breeders primarily raise large-sized Podenco Andaluz dogs and can be classified into three distinct typologies based on traditional breeding and selection practices, hunting area, and economic criteria.

The sustainability of hunting dog packs (rehalas) relies on the implementation of informed breeding and management strategies that integrate pedigree knowledge with the assessment of morphological and functional traits, while prioritizing animal welfare. Optimizing genetic selection may reduce the number of dogs required during driven hunts, thereby lowering economic costs and management pressure without compromising functional performance or welfare standards.

Beyond their practical role, dog packs constitute a cultural, genetic, and socio-ecological asset deeply embedded in Mediterranean rural territories. Their conservation requires policies that recognize the diversity of existing management typologies and promote welfare-oriented, context-specific practices, ensuring the long-term continuity of this traditional system and its contribution to sustainable rural development.

Future research should build on these findings by incorporating objective performance and welfare indicators. The use of GPS tracking and heart rate monitoring could allow the quantification of functional performance, spatial behavior, and physiological effort during hunting activities. In addition, genomic approaches, such as DNA sequencing, could be employed to assess whether long-term traditional selection by breeders has resulted in distinct genetic signatures when compared to other Mediterranean hunting dog populations. Finally, given the proximity of many dog pack facilities to urban areas and protected natural spaces, further studies should explore potential conflicts or synergies between traditional hunting systems, expanding urban populations, and nature-based tourism, contributing to more integrated territorial management strategies.

## Figures and Tables

**Table 1:** Socioeconomic characteristics of dog packs in Sierra Morena, Cordoba.

Variable	Class	Absolute Frequency	Relative Frequency (%)	MCA
Geographic location	Córdoba and Cerro Muriano	15	50.00	*
	Hornachuelos and Villaviciosa de Córdoba	11	36.67	
	Hinojosa del Duque, Azuel, La Rambla and Pedro Abad	4	13.33	
Educational level	None	1	3.33	*
	Primary	20	66.67	
	Secondary	4	13.33	
	Vocational	3	10.00	
	University	2	6.67	
Professional	Yes	30	100.00	
Occupation	Secondary	30	100.00	
Sex	Man	30	100.00	
Hunting area	Sierra Morena	26	86.67	*
	Sierra Morena and Ciudad Real	2	6.67	
	Sierra Morena, Ciudad Real and Los Alcornocales	2	6.67	
Usual prey	Rabbit	2	6.67	*
	Rabbit and wild boar	2	6.67	
	Ungulates	26	86.67	
Dog sex preference	No	10	33.33	*
	Male	15	50.00	
	Female	5	16.67	
Is financially supported ^1^	Yes/No	14	46.67	*
Type of services offered	Montería	30	100.00	
Type of dog	Podenco Campanero	4	13.33	*
	Tracking	4	13.33	
	Campanero, holding, tracking	6	20.00	
	Campanero, holding	14	46.67	
	Campanero–Mastiff cross, holding, tracking	1	3.33	
	Campanero, Campanero–Mastiff cross, holding	1	3.33	

^1^ Represents the number of affirmative answers. * Variables used for multiple correspondence analysis (MCA).

**Table 2:** Management and care characteristics of dog packs in Sierra Morena, Cordoba.

Variable	Class	Absolute Frequency	Relative Frequency (%)	MCA
Trains ^1^	Yes/No	29	96.67	
Trains by age groups ^1^	Yes/No	12	40.00	*
Trains in hunting group ^1^	Yes/No	14	46.67	*
Person who trains	Breeder	30	100.00	
Uses reinforcement/punishment ^1^	Yes/No	0	0.00	
Collaborates with other dog pack ^1^	Yes/No	18	60.00	*
Formation of dog pack team	No	4	13.33	*
	Yes, by hunting/terrain/species/other dog packs	5	16.67	
	Yes, by hunting/terrain/species	10	33.33	
	Yes, by terrain/species	5	16.67	
	Yes, by species	3	10.00	
	Yes, by hunting/terrain	1	3.33	
	Yes, by hunting/species	2	6.67	
Choosing dogs for hunting	Based on previous criteria	5	16.67	
	Personal criteria	17	56.67	
	Based on prey and fatigue	3	10.00	
	Based on prey	2	6.67	
	Based on fatigue	2	6.67	
	Best dogs	1	3.33	
Hunt with females in heat ^1^	Yes/No	0	0.00	
Neuters non-breeding males ^1^	Yes/No	4	13.33	
Gives animals to others ^1^	Yes/No	8	26.67	
Introduced external genetics ^1^	Yes/No	22	73.33	*
Last genetic introduction	<5 years	10	45.45	
	5 years	5	22.73	
	>5 years	7	31.82	
Daily assistance	None	8	26.67	*
	Family	11	36.67	
	Co-worker	8	26.67	
	Assistant	2	6.67	
	Employee	1	3.33	
Diet of dogs	Commercial feed	8	26.67	*
	Commercial feed, meat, game scraps, food scraps	3	10.00	
	Commercial feed, food scraps	2	6.67	
	Commercial feed, meat, bread	10	33.33	
	Commercial feed, meat	4	13.33	
	Commercial feed, bread	2	6.67	
	Meat, bread	1	3.33	
Provides game remains ^1^	Yes/No	3	10.00	
Vaccination plan	Rabies only	2	6.67	
	Puppies + rabies	27	90.00	
	Puppies + adult + rabies	1	3.33	
Incidence of leishmaniasis ^1^	Yes/No	13	43.33	*
Measures against leishmaniasis	None	15	50.00	*
	Collar	3	10.00	
	Cleaning + disinfection	10	33.33	
	Collar + insect traps + vaccination	1	3.33	
	Disinfection + insect traps	1	3.33	

^1^ Represents the number of affirmative answers. * Variables used for multiple correspondence analysis (MCA).

**Table 3:** Selection criteria for choosing breeding dogs of dog packs in Sierra Morena, Cordoba.

Variable	Class	Absolute Frequency	Relative Frequency (%)	MCA
External criteria	General type	12	40.00	*
	Regional type (body)	1	3.33	
	Size	1	3.33	
	Regional type (body) + coat	1	3.33	
	General type + regional type (body + limbs)	1	3.33	
	Regional type (head) + coat	1	3.33	
	General type + coat	2	6.67	
	General type + regional type (body)	3	10.00	
	General type + size	2	6.67	
	General type + coat + size	1	3.33	
	General type + regional type (head, body, limbs)	2	6.67	
	General type + regional type (head/body) + coat + size	1	3.33	
	General type + regional type (body) + coat	1	3.33	
	General type + head + coat + size	1	3.33	
Functional criteria	None	1	3.33	*
	Endurance, grip, intelligence	5	16.67	
	Endurance, olfaction, intelligence	2	6.67	
	Endurance, obedience	1	3.33	
	Latency, intelligence	2	6.67	
	Endurance, latency	5	16.67	
	Endurance, grip, intelligence, latency, strength	1	3.33	
	Endurance, grip, intelligence, latency	3	10.00	
	Endurance, grip, olfaction, latency	2	6.67	
	Endurance, grip	1	3.33	
	Endurance, olfaction, latency	3	10.00	
	Endurance, grip, latency	1	3.33	
	Endurance, latency, strength	3	10.00	
Temperament criteria	None	2	6.67	*
	Nobility	16	53.33	
	Bravery	4	13.33	
	Distrust	1	3.33	
	Maternal aptitude	1	3.33	
	Nobility + bravery	5	16.67	
	Nobility + bravery + distrust	1	3.33	

* Variables used for multiple correspondence analysis (MCA).

**Table 4:** Descriptive statistics of dog packs in Sierra Morena, Cordoba.

Variable	Mean	Coefficient of Variation	Standard Error	Minimum	Maximun	MCA *
Number of animals	51.87	36.85	3.49	25.00	106.00	*
Number of males	39.57	41.77	3.02	15.00	92.00	
Number of females	12.23	36.35	0.81	6.00	25.00	
Number of tracking dogs	48.63	37.18	3.30	25.00	106.00	
Number of running dogs	48.63	37.18	3.30	25.00	106.00	
Number of holding dogs	16.70	135.89	4.14	1.00	106.00	
Number of caretakers	1.40	44.39	0.11	1.00	3.00	*
Average hunts per year	39.67	58.73	4.25	5.00	130.00	*
Days animals train	2.93	57.97	0.31	0.00	7.00	*
Days dog pack trains	2.70	48.78	0.24	0.00	7.00	
Training time (h/week)	2.85	36.90	0.19	0.00	6.00	*
Age at start of training (months)	10.45	34.92	0.67	4.00	18.00	*
Age of dog pack owner (years)	48.00	25.19	2.21	25.00	68.00	*
Years of experience	28.50	44.57	2.32	3.00	60.00	
Seasons before becoming breeder	1.83	60.91	0.20	1.00	4.00	*
Deworming schedule (months)	6.07	42.62	0.47	3.00	12.00	*

* Variables used for multiple correspondence analysis (MCA).

**Table 5:** Factors of multiple correspondence analysis of dog packs in Sierra Morena, Cordoba.

Variable	Loading	Eigenvalue	Explained Variance (%)	Acumulate (%)	Factor
Average number of hunts per year	0.94	4.59	16.40	16.40	1
Number of animals	0.86				1
Type of dog pack	0.54				1
Hunting area	−0.93	3.81	13.61	30.02	2
Age of dog pack owner (years)	0.53				2
Training time (h/week)	0.95	2.76	9.87	39.89	3
Diet provided	−0.89	2.40	8.58	48.47	4
Usual prey	0.62				4
Municipality	0.91	2.23	7.96	56.42	5
Deworming plan (months)	0.96	1.83	6.55	62.97	6
Collaboration with other dog pack	−0.87	1.74	6.23	69.20	7
Temperament criteria	0.94	1.49	5.32	74.52	8
Training in hunting group	0.54				8
Incidence of leishmaniasis	−0.94	1.30	4.63	79.15	9
Days animals train	0.90	0.90	3.22	82.37	10

**Table 6:** Characteristics and comparative analysis of three dog pack typologies. In brackets is the number of pack dogs.

Variable	Clase	Cluster			*p*_Value ^1^
		1 (9)	2 (5)	3 (16)	
Municipality	Córdoba and Cerro Muriano	44.44	40	56.25	n.s.
	Hornachuelos and Villaviciosa	55.56	60	18.75	
	Azuel, Hinojosa del Duque, La Rambla and Pedro Abad	0	0	25	
Educational level	Primary	66.67	100	56.25	n.s.
	Secondary	22.22	0	12.50	n.s.
	Vocational	0	0	18.75	n.s.
	University	11.11	0	6.25	n.s.
Age of dog pack owner (years)		50.67 ± 4.63	49.40 ± 5.31	46.06 ± 2.89	n.s.
Hunting area	Sierra Morena	88.89	100.00	75.00	n.s.
Usual prey	Ungulates	77.78	80.00	87.50	n.s.
Dog sex preference	Male	55.56	20	56.25	n.s.
Is financially supported	Yes	33.33	80	43.75	n.s.
Type of dog	Campanero and crosses	77.78	80.00	93.75	n.s.
Number of animals		44.67 ± 4.59	45.80 ± 6.61	57.81 ± 5.39	n.s.
Number of caretakers		1.44 ± 0.18	1.00 ± 0.00	1.50 ± 0.18	n.s.
Average number of hunts per year		32.56 ± 6.30	36.00 ± 6.20	44.81 ± 6.82	n.s.
Days animals train		2.33 ± 0.17	3.60 ± 0.81	3.06 ± 0.51	n.s.
Training time (h/week)		3.00 ± 0.22	3.10 ± 0.78	2.69 ± 0.25	n.s.
Age at start of training (months)		11.11 ± 1.26	11.20 ± 0.80	9.84 ± 1.01	n.s.
Trains by ages group	Yes	44.44	40.00	31.25	n.s.
Trains in hunting group	Yes	55.56	60.00	37.50	n.s.
Collaborate with other dog pack	Yes	55.56	100	50.00	n.s.
Formation of dog pack team	Yes	77.77	100	93.75	n.s.
Introduced external genetics	Yes	77.78	60.00	68.75	n.s.
Daily assistance	Yes	77.77	60.00	87.50	n.s.
Diet of dog	Commercial feed	33.33	40.00	18.75	n.s.
Deworming schedule (months)		6.00 ± 0.82	9.20 ± 1.74	5.13 ± 0.30	*p* < 0.01
Incidence of leishmaniasis	Yes	55.56	20.00	43.75	n.s.
Measures against leishmaniasis	Yes	33.33	60.00	56.25	n.s.

^1^ n.s. = no significant differences at level of *p* > 0.05.

## Data Availability

The data presented in this study are not publicly available due to ethical and confidentiality considerations, but are available upon reasonable request from the corresponding author.
